# Supplementary material for: MetaQTL: a package of new computational methods for the meta-analysis of QTL mapping experiments
Source: BMC Bioinformatics. 2007 Feb 8;8:49. doi: 10.1186/1471-2105-8-49 (PMC1808479; doi:10.1186/1471-2105-8-49)
Supplement: Additional File 7 — MetaQTL Package : jar file and tutorial. This Zip archive contains both the MetaQTL JAR file and the files of the tutorial. [file 1471-2105-8-49-S7.zip › org.inra.metaqtl/doc/org/metaqtl/bio/util/package-frame.html]

org.metaqtl.bio.util


org.metaqtl.bio.util

Interfaces 
  
*ILGroupPosition*

Classes 
  
BioCrossTypes
  
BioEntityNameComparator
  
BioLocusTypes
  
BioUtilities
  
EstimatedLocusPosition
  
LGroupPosition
  
LocusPositionComparator
  
NumberFormat
  
StaticLocusPosition
  
TabulatedReader
  
TabulatedWriter
